# Predictability, Skeletal Stability, and Safety of Iliac Crest Bone Grafts in Large Maxillary Advancement with Le Fort I Osteotomy: A Systematic Review

**DOI:** 10.3390/jcm15072586

**Published:** 2026-03-28

**Authors:** Kamil Nelke, Agnieszka Kotela, Zuzanna Majchrzak, Kamil Wesołek, Agata Małyszek, Marzena Laszczyńska, Jacek Matys, Maciej Dobrzyński

**Affiliations:** 1 Maxillo-Facial Surgery Ward, EMC Hospital, Pilczycka 144, 54-144 Wrocław, Poland; kamil.nelke@gmail.com; 2 Academy of Applied Sciences, Health Department, Academy of Silesius in Wałbrzych, Zamkowa 4, 58-300 Wałbrzych, Poland; 3 Medical Center of Innovation, Wroclaw Medical University, Krakowska 26, 50-425 Wrocław, Polandzuzanna.h.nawrocka@gmail.com (Z.M.);; 4 Department of Biostructure and Animal Physiology, Wroclaw University of Environmental and Life Sciences, Kozuchowska 1, 51-631 Wrocław, Poland; 5 Dental Surgery Department, Wroclaw Medical University, Krakowska 26, 50-425 Wrocław, Poland; 6 Department of Pediatric Dentistry and Preclinical Dentistry, Wroclaw Medical University, Krakowska 26, 50-425 Wrocław, Poland

**Keywords:** autogenous bone graft, iliac crest bone graft, large maxillary advancement, Le Fort I osteotomy, orthognathic surgery, skeletal stability

## Abstract

**Objective:** The aim of this systematic review was to evaluate the skeletal stability, predictability, and safety of using autogenous iliac crest bone grafts (ICBG) during large maxillary advancement performed with Le Fort I osteotomy. **Methods:** A systematic literature search was performed in November 2025 using PubMed, Scopus, Embase, Web of Science, and WorldCat databases. Clinical studies reporting large maxillary advancement performed with Le Fort I osteotomy and incorporating ICBG were included. Study selection followed PRISMA guidelines. Data extraction focused on the magnitude of maxillary advancement, surgical protocols, stabilization methods, skeletal stability, relapse patterns, graft integration, implant-related outcomes, and complications. Methodological quality was assessed using the Mixed-Methods Appraisal Tool (MMAT). **Results:** The review included clinical studies predominantly consisting of case reports, case series, and retrospective cohort studies. ICBG were consistently used in complex clinical scenarios, such as severe maxillary atrophy, hypoplasia, and congenital craniofacial deformities. Large maxillary advancements were generally associated with favorable postoperative skeletal stability, with most relapse occurring during the early healing phase and minimal changes observed during long-term follow-up when rigid fixation and adequate graft integration were achieved. Interpositional grafting facilitated predictable advancement by bridging extensive osteotomy gaps. Donor-site morbidity related to iliac crest harvesting was typically mild and transient. Implant-related outcomes, reported as secondary findings, were generally favorable when implants were placed after an adequate healing period. **Conclusions:** Despite predominantly observational evidence, ICBG during large maxillary advancement with Le Fort I osteotomy appears to offer predictable advancement, acceptable skeletal stability, and a favorable safety profile, warranting further prospective investigation.

## 1. Introduction

The management of skeletal malocclusions frequently requires a combination of orthodontic and surgical interventions, with the extent of skeletal discrepancy determining the complexity of the surgical approach. In cases characterized by advanced bone deficiency, the use of bone grafting materials has been proposed to compensate for osteotomy gaps, support bone healing, and enhance postoperative skeletal stability [1,2,3,4,5,6,7,8,9]. Contemporary trends in orthognathic surgery increasingly emphasize the closure of bone gaps and restoration of adequate bone volume and contour at osteotomy sites to optimize functional and aesthetic outcomes [10]. Bone grafting has therefore been applied in syndromic and non-syndromic deformities, as well as in asymmetric conditions, to re-establish facial skeletal balance and provide adequate support for the overlying soft tissues [10,11].

A wide range of grafting materials is currently available in orthognathic surgery, including autologous, allogenic, and xenogenic bone substitutes, as well as alloplastic materials [12,13,14,15,16]. The indication for bone grafting and the selection of graft type depend on multiple interrelated factors, such as the size and configuration of the osteotomy gap, the extent of the underlying skeletal abnormality, and the biological limits of spontaneous bone healing [17,18,19,20,21,22,23]. Although xenografts and allogenic grafts offer advantages in terms of availability and reduced donor-site morbidity, their biological behavior, structural characteristics, and integration potential may be limited in extensive skeletal defects, particularly when large, unsupported gaps are created [24]. These limitations become especially relevant in the context of large maxillary advancements and vertical repositioning, where sagittal and/or vertical movements may result in wide osteotomy gaps and reduced bony contact between segments [25,26,27,28,29,30,31,32,33,34,35]. In such situations, the natural healing capacity of the maxilla may be exceeded, potentially compromising early postoperative stability and increasing the risk of unfavorable skeletal changes. Clinical uncertainty therefore persists regarding the necessity and effectiveness of bone grafting when maxillary advancement or extrusion exceeds approximately 5 mm, a threshold often considered biomechanically demanding in orthognathic surgery [24]. In the present review, large maxillary advancement was defined as maxillary advancement and/or downgrafting greater than 5 mm, or as extensive maxillary repositioning described by the authors as resulting in a clinically significant bone gap and insufficient bony contact between the mobilized segments, often requiring additional bone support.

**Figure 1:**
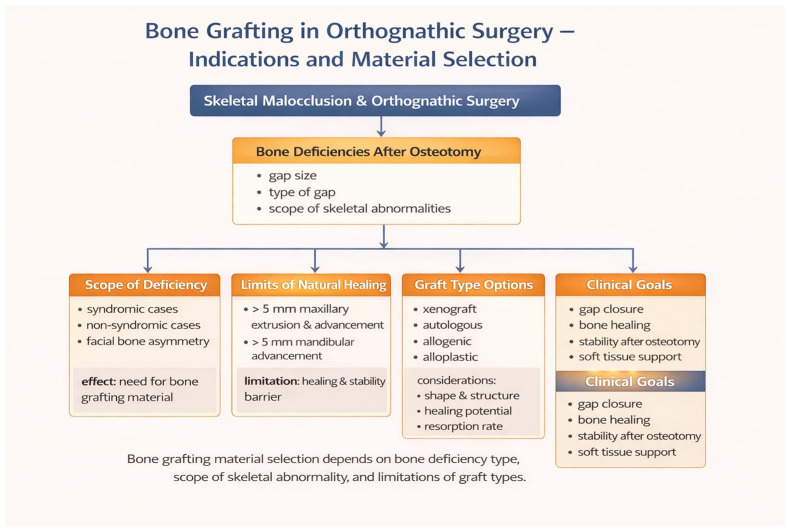
Indications for bone grafting and material selection in orthognathic surgery.

In this setting, bone grafting is frequently considered not only to facilitate gap closure and bone healing but also to enhance skeletal stability and provide adequate support for the overlying soft tissues, as conceptually summarized in Figure 1.

Autogenous bone grafts harvested from intraoral or regional donor sites may be sufficient for smaller defects; however, their volume and geometry can be inadequate when extensive maxillary advancement or downgrafting is required [24]. In such situations, autogenous iliac crest bone grafting (ICBG) has historically been advocated as a reliable source of both cortical and cancellous bone, providing structural support, biological potential for remodeling, and compatibility with rigid fixation systems [36]. The application of ICBG has been particularly emphasized in patients with severe vertical deficiency, short-face syndrome, or complex craniofacial deformities, where large sagittal and/or vertical maxillary repositioning is necessary to restore occlusion, facial proportions, and incisor exposure [37,38,39,40,41].

Despite these potential advantages, the use of ICBG introduces additional considerations, including donor-site morbidity, extended operative time, and the need for a second surgical field [42]. These factors may influence surgical decision-making and raise questions regarding the balance between improved skeletal predictability and procedural burden [43,44,45,46,47,48,49,50,51,52,53,54]. While ICBG has been widely adopted in complex reconstructive scenarios, the evidence supporting its role specifically in large maxillary advancement performed with Le Fort I osteotomy remains heterogeneous and largely based on observational data.

Accordingly, the present systematic review aimed to evaluate the skeletal stability, predictability, and safety associated with the use of ICBG during large maxillary advancement performed with Le Fort I osteotomy, synthesizing the available clinical evidence to clarify their role in contemporary orthognathic and reconstructive practice.

## 2. Materials and Methods

### 2.1. Focused Question

This systematic review was conducted in accordance with the Preferred Reporting Items for Systematic Reviews and Meta-Analyses (PRISMA) guidelines (PRISMA Checklist can be seen in the Supplementary Materials), and the focused research question was developed using the PICO framework as follows:

Population (P): Patients undergoing Le Fort I osteotomy involving large maxillary advancement, defined as maxillary advancement and/or downgrafting greater than 5 mm, or described by the authors as extensive maxillary repositioning resulting in a clinically significant osteotomy gap and insufficient bony contact between the mobilized segments, often requiring additional bone support.

Intervention (I): Use of ICBG applied during Le Fort I osteotomy, either as interpositional or onlay grafts.

Comparator (C): Not mandatory, as most available evidence consists of observational studies without a control group.

Outcomes (O): Postoperative skeletal stability of the maxilla, predictability of achieving large maxillary advancement, and safety of the procedure, including relapse patterns and graft-related or donor-site complications, assessed using cephalometric, radiological, and clinical evaluations.

Based on this framework, the focused research question was formulated as follows: What is the skeletal stability, predictability, and safety of using ICBG during large maxillary advancement performed with Le Fort I osteotomy?

### 2.2. Protocol

**Figure 2:**
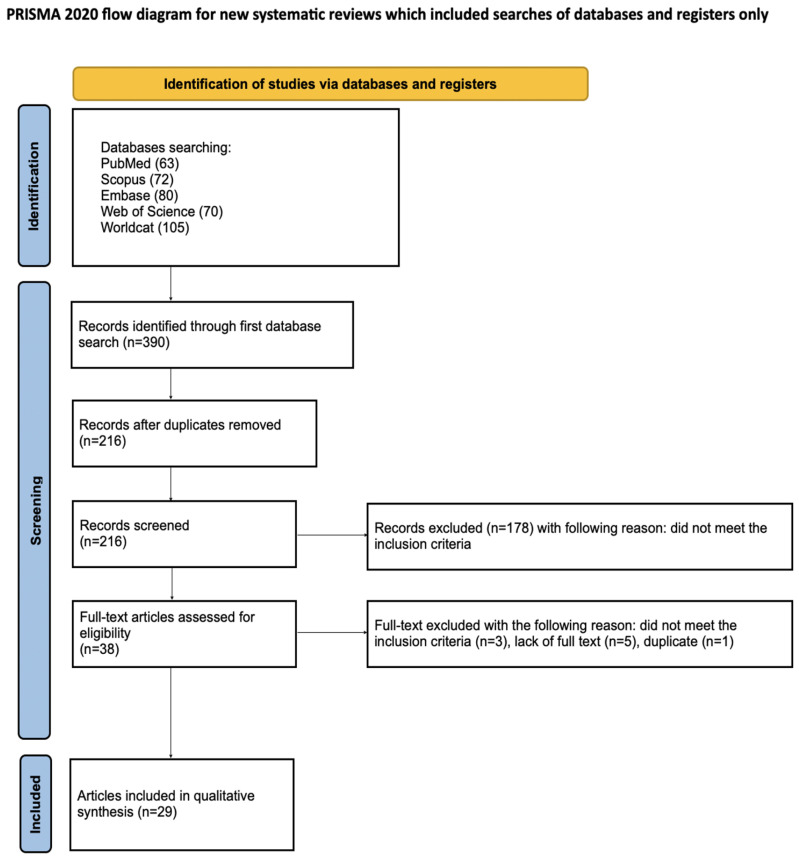
PRISMA flow diagram [55].

The process of study identification, screening, and selection was predefined and conducted in accordance with the PRISMA 2020 flow diagram (Figure 2) [25]. The review protocol was registered prospectively in the Open Science Framework, and the registration record is available online https://doi.org/10.17605/OSF.IO/47NFB (accessed on 8 February 2026).

### 2.3. Eligibility Criteria

Interventionary studies involving animals or humans, and other studies that require ethical approval, must list the authority that provided approval and the corresponding ethical approval code. Studies were considered eligible for inclusion in this systematic review if they fulfilled all of the following criteria: [56,57,58,59,60,61,62,63,64,65].

- Included patients undergoing Le Fort I osteotomy involving large maxillary advancement, defined as maxillary advancement and/or downgrafting greater than 5 mm, or described by the authors as extensive maxillary repositioning associated with a clinically significant osteotomy gap and insufficient bony contact between the mobilized segments, often requiring additional bone support;
- Reported the use of ICBG applied during the surgical procedure, either as interpositional or onlay grafts;
- Were clinical studies, including randomized controlled trials, prospective or retrospective cohort studies, and case series;
- Were published in English;
- Provided full-text access to allow detailed data extraction.

Studies were excluded from the review if they met any of the following criteria: [56,57,58,59,60,61,62,63,64,65].

- Did not involve the use of ICBG during Le Fort I osteotomy;
- Were non-English publications;
- Were conference abstracts, editorials, narrative or systematic reviews, or technical notes without original clinical data;
- Lacked accessible full-text versions;
- Represented duplicate publications or overlapping patient populations, in which case only the most comprehensive or recent report was included.

No restrictions were applied regarding the year of publication.

### 2.4. Information Sources, Search Strategy, and Study Selection

A comprehensive electronic literature search was performed in November 2025 using five databases: PubMed, Scopus, Embase, Web of Science (WoS), and WorldCat. The search strategy was designed to identify clinical studies addressing the use of ICBG in the context of large maxillary advancement performed with Le Fort I osteotomy. Searches were restricted to titles and abstracts to enhance specificity.

The detailed search strategies applied in each database were as follows:

- PubMed: (“Le Fort I” [Title/Abstract] OR “LeFort I” [Title/Abstract]) AND (“iliac crest” [Title/Abstract] OR autogenous” [Title/Abstract] OR “corticocancellous” [Title/Abstract]);
- Scopus: TITLE-ABS (“Le Fort I” OR “LeFort I”) AND TITLE-ABS (“iliac crest” OR “autogenous” OR “corticocancellous”);
- Embase: (‘le fort i’:ab,ti OR ‘lefort i’:ab,ti) AND (‘iliac crest’:ab,ti OR autogenous:ab,ti OR corticocancellous:ab,ti);
- Web of Science (WoS): TS = (“Le Fort I” OR “LeFort I”) AND TS = (“iliac crest” OR “autogenous” OR “corticocancellous”);
- WorldCat: (“Le Fort I” OR “LeFort I”) AND (“iliac crest” OR “autogenous” OR “corticocancellous”). WorldCat was additionally searched as a supplementary source to identify potentially relevant books, monographs, dissertations, and older records not indexed in standard biomedical databases, particularly given the historical development of surgical techniques related to this topic.

All records retrieved through the database searches were screened in accordance with the predefined eligibility criteria. Following the removal of duplicates, titles and abstracts were independently assessed, and only studies with accessible full-text versions were considered for inclusion in the final qualitative synthesis.

### 2.5. Data Collection Process and Data Items

Four independent reviewers (A.K., M.L., Z.M., and K.W.) screened the eligible studies and performed data extraction using a predefined protocol. For each included study, the following data were collected: first author and year of publication, study design, number of patients, magnitude of maxillary advancement, type and configuration of the ICBG, duration of follow-up, primary outcomes related to skeletal stability and predictability, and reported graft-related or donor-site complications.

All extracted data were systematically compiled in a standardized Microsoft Excel spreadsheet to ensure consistency and accuracy during analysis.

### 2.6. Risk of Bias and Quality Assessment

To minimize selection bias, all reviewers independently screened titles and abstracts of retrieved records. Inter-reviewer agreement during the screening process, assessed using Cohen’s kappa coefficient, was substantial (κ = 0.78). Discrepancies regarding study inclusion or exclusion were resolved through consensus-based discussion among the authors.

### 2.7. Quality Assessment

The methodological quality of the included studies was independently assessed by two blinded reviewers (J.M. and M.D.) using the Mixed-Methods Appraisal Tool (MMAT), version 2018. The MMAT is a validated instrument designed to evaluate the methodological rigor of studies employing different research designs, including randomized, non-randomized, descriptive quantitative, and mixed-methods approaches. The following guiding questions were used in the appraisal process:

- Is the sampling strategy relevant to address the research question?
- Is the sample representative of the target population?
- Are the measurements appropriate?
- Is the risk of nonresponse bias low?
- Is the statistical analysis appropriate to answer the research question?

Each study was appraised according to five predefined MMAT criteria addressing the appropriateness of the sampling strategy, representativeness of the study population, adequacy of outcome measurements, risk of nonresponse bias, and suitability of the statistical analysis. Each criterion was rated as “yes,” “no,” or “can’t tell.” Disagreements between reviewers were resolved through consensus-based discussion. Inter-rater reliability was quantified using Cohen’s kappa coefficient, calculated with MedCalc software (version 23.1.7; MedCalc Software Ltd., Brussels, Belgium). The resulting kappa value (κ = 0.85; *p* < 0.001) indicated near-perfect agreement between reviewers.

## 3. Results

### 3.1. Study Selection

Study selection process aimed to identify clinical reports evaluating the use of ICBG during Le Fort I osteotomy performed for large maxillary advancement. Studies were screened based on their relevance to maxillary repositioning requiring substantial sagittal and/or vertical advancement in which ICBG were used to facilitate reconstruction and enhance postoperative stability. Publications were excluded if Le Fort I osteotomy was not performed, if ICBG were not used, or if the reported procedures did not address clinically meaningful maxillary advancement. Studies focusing exclusively on isolated complications without reporting reconstructive outcomes were also excluded. Clinical reports, including case reports, case series, retrospective cohort studies, and studies with long-term follow-up, were considered eligible when they documented outcomes related to skeletal stability, predictability, or safety following large maxillary advancement using ICBG [66,67,68,69,70,71,72,73,74,75,76,77,78,79,80,81,82,83,84,85,86,87,88,89,90]. Following the screening and eligibility assessment, a final group of studies was included that specifically addressed the role of ICBG in achieving and maintaining large maxillary advancements performed with Le Fort I osteotomy.

### 3.2. General Characteristic of Included Studies

The included studies comprised a heterogeneous body of clinical evidence describing extensive maxillary advancements performed with Le Fort I osteotomy combined with ICBG. The majority of publications were observational in nature and reflected the complexity of patients requiring large maxillary repositioning. In terms of study design, the included literature consisted predominantly of case reports (*n* = 6; [66,69,70,71,87,91]), case series (*n* = 12; [67,72,73,74,76,77,78,82,83,84,92,93]), and retrospective cohort studies (*n* = 7; [68,79,80,81,85,86,89]). Additionally, a limited number of prospective clinical studies or randomized investigations were identified (*n* = 2; [75,88]). Several studies provided long-term follow-up data exceeding five years, with some extending beyond ten years [80,82]. Most studies included patients with severe maxillary atrophy, advanced maxillary hypoplasia, or congenital craniofacial conditions, such as cleft lip and palate, ectodermal dysplasia, or other craniofacial syndromes [66,69,73,74,85,89,90]. These conditions typically necessitated extensive sagittal and/or vertical maxillary advancement beyond conventional orthognathic movements.

ICBG was consistently used as the primary grafting material across all included studies, most commonly in the form of corticocancellous block grafts placed interpositionally to fill large osteotomy gaps or as onlay grafts to restore maxillary contour and volume [67,68,71,72,76,77,79,80,82,92,93]. Both single-stage and two-stage surgical protocols were reported. In cases involving extensive reconstruction, delayed implant placement was frequently employed, most commonly after a healing period of approximately 4–6 months following the initial surgery [72,73,76,77,78,82,92]. Fixation techniques varied among studies and included rigid internal fixation with plates and screws, as well as wire osteosynthesis, depending on the surgical approach, period of publication, and underlying pathology [86]. Follow-up duration ranged from short-term postoperative assessment to long-term observation, allowing evaluation of skeletal stability, graft integration, and functional rehabilitation after large maxillary advancement [80,82].

A detailed summary of study characteristics is presented in Table 1.

### 3.3. Main Study Outcomes

#### 3.3.1. Predictability of Large Maxillary Advancement

Across the included studies, ICBG were reported as a key component in enabling large maxillary advancements performed with Le Fort I osteotomy, particularly in patients with severe maxillary atrophy, hypoplasia, or complex congenital deformities [67,71,72,73,76,77,78,79,80,82,92,93]. In these reports, ICBG were used to bridge extensive osteotomy gaps and to provide structural support when conventional bone contact between segments was insufficient. Studies describing more substantial sagittal and/or vertical advancements emphasized the role of interpositional grafting in maintaining maxillary position during the early healing phase and facilitating predictable advancement in cases exceeding conventional orthognathic limits [71,78,79,85]. Reports involving extreme advancements were limited to selected case series and individual case reports, reflecting highly specialized reconstructive indications rather than routine orthognathic procedures [93].

#### 3.3.2. Skeletal Stability and Relapse Patterns

Postoperative skeletal stability following large maxillary advancement with ICBG was generally favorable. Several studies reported that the majority of skeletal relapse occurred during the early postoperative period, typically within the first 3 to 6 months after surgery [71,78,79,80,85]. After this initial phase, positional changes of the maxilla were minimal, provided that rigid internal fixation and adequate graft integration had been achieved [78,79,80,82]. Quantitative analyses demonstrated that horizontal relapse was generally limited, often ranging between approximately 1 and 2 mm, while vertical relapse was less pronounced and showed greater variability among studies [78,79,85,86]. Long-term follow-up data extending beyond five years confirmed sustained skeletal stability with no clinically significant late relapse in the majority of cases [72,80,82,93]. Studies focusing on cleft-related maxillary advancement reported early relapse followed by long-term stabilization, without a significant correlation between the magnitude of advancement and the degree of relapse [90].

#### 3.3.3. Graft Integration and Bone Remodeling

Successful incorporation of ICBG was reported in most included studies. Radiographic and clinical assessments demonstrated progressive graft integration and remodeling over time, particularly when interpositional grafts were used to fill large osteotomy gaps [71,72,73,76,78,80,82,92]. Early graft resorption was observed in some cases but generally stabilized after the initial healing period, with long-term bone volume remaining sufficient to support maxillary stability and subsequent rehabilitation [80,82,93].

#### 3.3.4. Implant-Related Outcomes

Implant-related outcomes were reported as secondary findings in a subset of studies. When dental implants were placed in grafted maxillae following an adequate healing period, typically ranging from 4 to 6 months after surgery, implant survival rates were generally favorable [76,80,82,84,92]. Early implant failures were infrequently reported and were mainly associated with insufficient primary stability or premature loading, whereas delayed implant placement was associated with improved osseointegration [77,78,83].

#### 3.3.5. Safety and Complications

Donor-site morbidity related to iliac crest bone harvesting was reported in several studies and was generally mild and transient, consisting primarily of postoperative pain or temporary gait disturbance that resolved without long-term sequelae [68,76,79,80,94]. Complications at the recipient site were uncommon and included occasional soft tissue dehiscence, minor graft resorption, or sinus-related complications, most of which were managed conservatively or with minor secondary interventions [72,73,82,92,93]. Serious complications were rare and were typically associated with complex syndromic cases or combined surgical procedures [74,91].

Generally, the included studies indicate that the use of ICBG during large maxillary advancement with Le Fort I osteotomy is associated with predictable advancement, favorable skeletal stability, and an acceptable safety profile (see Table 2).

### 3.4. Quality Assessment

Assessment using the five MMAT criteria demonstrated variability in the methodological quality of the included studies. Fifteen studies fulfilled all five MMAT criteria [72,73,78,79,80,81,82,83,84,87,88,89,92,93,94]. Six studies met four of the five criteria [74,76,77,85,86,90], while two studies fulfilled three criteria [68,75]. The remaining six studies met two of the five criteria [66,67,69,70,71,91], consisting predominantly of case reports and very small case series. The most frequently unmet criteria were sample representativeness and appropriateness of statistical analysis, reflecting the observational and descriptive nature of much of the available evidence. A detailed overview of the MMAT assessment is provided in Table 3.

## 4. Discussion

The present systematic review reinforces a critical perspective on large maxillary advancements performed with the Le Fort I osteotomy, particularly in relation to the biomechanical limitations of conventional orthognathic movements. Previous studies have suggested that extensive sagittal and vertical repositioning of the maxilla may be associated with an increased risk of early postoperative relapse, especially in patients with severe maxillary atrophy, cleft-related deformities, or syndromic conditions [48,50,51,52,53,54,85]. However, the findings of the present review indicate that relapse following large maxillary advancement predominantly occurs during the early healing phase and does not necessarily progress during long-term follow-up [95]. In this context, the available literature supports the use of ICBG as a stabilizing adjunct that facilitates extensive maxillary advancement while maintaining acceptable long-term skeletal outcomes [77]. Nevertheless, the predominance of case reports and retrospective cohort studies among the included publications [67,71,72,73,76,79,80,82] highlights the lack of standardized criteria regarding advancement magnitude, fixation methods, and outcome assessment, which limits direct comparability between studies.

From a reconstructive standpoint, ICBG appear to function both as structural supports and biologically active scaffolds, reducing mechanical strain on fixation systems and promoting bone regeneration within large osteotomy gaps [96,97]. Several authors have emphasized that interpositional grafting is particularly relevant in vertical augmentation and combined anteroinferior movements, where unsupported maxillary repositioning has been reported to be less predictable [98]. Consistent with the results of the present review, long-term follow-up data indicate that when sufficient healing periods are allowed, implant rehabilitation in grafted maxillae can achieve favorable outcomes; however, early implant failure and variable remodeling remain potential concerns and should be interpreted as secondary findings rather than primary endpoints [77]. Donor-site morbidity associated with iliac crest harvesting was frequently reported but was generally transient and did not outweigh the reconstructive benefits in complex maxillary deficiencies [99]. Overall, the reviewed evidence supports ICBG as a biologically and mechanically advantageous adjunct in selected cases of large Le Fort I advancement rather than as a routine intervention.

In this review, large maxillary advancement was defined as movements of 5 mm or greater performed with Le Fort I osteotomy. Across the included studies, such advancements were most frequently reported in clinically complex situations, including severe maxillary atrophy, hypoplasia, or congenital craniofacial conditions [66,67,69,71,72,73,76,77,78,79,80,82,85,92,93,94]. Movements of this magnitude are clinically relevant because they often result in a wide osteotomy gap and reduced bony contact between segments, which may compromise early postoperative stability [71,78,79,85]. Notably, all studies included in this review incorporated bone grafting as part of the surgical protocol for large maxillary advancement. This consistent application reflects prevailing clinical practice within the available evidence base rather than comparative effectiveness and suggests that bone grafting is widely regarded as a justified strategy for managing extensive osteotomy gaps in movements of 5 mm or greater [67,71,72,73,76,77,78,79,80,82,92,93]. Studies included in the present review reported that insufficient stabilization was associated with greater early postoperative skeletal changes, whereas reconstructive protocols incorporating bone grafting demonstrated more predictable outcomes with limited relapse during follow-up [71,78,79,80,85,90]. Similar considerations have been reported in earlier clinical investigations and broader analyses of orthognathic surgery, which describe large maxillary movements as biomechanically demanding and emphasize the importance of maintaining stability during the early postoperative period [77,85,100,101,102,103,104,105].

Across the analyzed studies, the surgical protocol and stabilization strategy represented a critical component of clinical management in patients undergoing large maxillary advancement with Le Fort I osteotomy and ICBG. The included reports consistently described stabilization as an integral element of the reconstructive approach accompanying extensive sagittal and/or vertical repositioning of the maxilla. Both single-stage and two-stage surgical protocols were employed, reflecting differences in reconstructive complexity and clinical indication within the analyzed material [72,73,76,77,78,82,92]. Stabilization of the advanced maxilla was most commonly achieved using rigid internal fixation with plates and screws, whereas wire osteosynthesis was reported in selected studies, particularly in earlier series [86,90]. Several reports additionally described rigid fixation of the graft at the osteotomy site to maintain segment position during the early healing period [71,72,78,79,82]. Collectively, the findings of the included studies indicate that clearly defined stabilization strategies are widely considered essential procedural factors in the surgical management of extensive maxillary repositioning

Several limitations of this review should be acknowledged. Although several studies reported quantitative findings, the available data were too heterogeneous in terms of relapse measurement, follow-up duration, and outcome definitions to permit meaningful quantitative synthesis or pooled analysis. The majority of the included studies were case reports, case series, and retrospective cohort studies, which introduce considerable heterogeneity in study design, patient populations, and outcome assessment. Most included studies were retrospective and involved relatively small patient cohorts, which reduced methodological consistency and the overall strength of the available evidence [66,67,68,69,70,71,72,73,74,76,77,78,79,80,81,82,83,84,85,86,87,89,90,91,92,93]. Considerable variability was observed in the reporting of maxillary advancement and postoperative stability, with displacement described in millimeters, by cephalometric parameters, or through descriptive clinical assessment, thereby reducing comparability across studies [78,79,80,85,86,90]. The included study populations were also clinically heterogeneous, ranging from isolated maxillary hypoplasia to severe atrophy and complex craniofacial deformities [66,69,73,74,85,89,90]. In addition, follow-up durations varied substantially between studies, complicating the assessment of long-term skeletal stability [72,78,79,80,82,93]. Within the current evidence base, these factors limit direct comparison between studies and preclude firm conclusions regarding the comparative effectiveness of ICBG. Future research should therefore focus on prospective study designs with standardized reporting of skeletal movement and relapse, as well as comparable follow-up intervals, particularly in patients undergoing extensive maxillary repositioning.

## 5. Conclusions

The available clinical evidence suggests that ICBG may be a useful adjunct in large maxillary advancement performed with Le Fort I osteotomy, particularly in complex cases involving severe maxillary atrophy, hypoplasia, or craniofacial deformities. Across the included studies, interpositional ICBG was generally associated with relatively predictable maxillary repositioning, generally favorable skeletal stability, and an acceptable safety profile, with most relapse occurring during the early postoperative healing period and limited long-term positional changes when rigid fixation and adequate graft integration were achieved. Implant-related outcomes were also generally favorable when implant placement was delayed to allow sufficient healing. However, the current evidence base is dominated by observational studies and is characterized by substantial heterogeneity in patient populations, surgical protocols, fixation methods, and outcome reporting. Therefore, these findings should be interpreted with caution. Within these limitations, ICBG appears to be a potentially beneficial option in selected cases of large Le Fort I advancement; however, its comparative effectiveness and long-term superiority cannot be established on the basis of the currently available evidence. Stronger prospective comparative studies with standardized definitions and outcome measures are needed to better define indications, optimize protocols, and confirm long-term effectiveness and safety.

## Figures and Tables

**Table 1:** General characteristics of included studies.

Study	Aim of the Study	Material and Methods	Results	Conclusions
Pombo Castro [66], 2013	To demonstrate oral rehabilitation of ectodermal dysplasia using combined preprosthetic bone grafting techniques and implant-supported prosthesis.	A 27-year-old male with ectodermal dysplasia, severe jaw atrophy and oligodontia. Two-stage surgical treatment: (1) Le Fort I osteotomy, bilateral sinus lift, and 7 bone grafts (5 onlay, 2 inlay) using ICBG with titanium fixation; (2) extraction of remaining teeth and placement of 11 Straumann implants (5 maxillary, 6 mandibular) six months later.	Partial graft exposure occurred due to smoking, requiring sequestra removal but sufficient bone remained for implant placement. All 11 implants successfully osseointegrated. At 2-year follow-up, all implants were stable with no failures.	Combined preprosthetic techniques (Le Fort I osteotomy, sinus lift, onlay/inlay grafts) with subsequent implant-supported fixed prosthesis is an effective treatment for oral rehabilitation in ectodermal dysplasia
Popat [91] 2024	To report a postoperative complication of air embolism-induced ischemic stroke following orthognathic surgery in a patient with Goldenhar syndrome	20-year-old male with Goldenhar syndrome underwent orthognathic surgery (LeFort I, BSSO, ICBG). EBL 500 cc. Developed postop right arm weakness. Imaging revealed left thalamic infarct with air in cavernous sinus/interpeduncular system, consistent with air embolism. Managed conservatively with neuro monitoring.	Imaging confirmed acute left thalamic infarct with air in cavernous sinus and interpeduncular system. 3-month follow-up: improvement, only residual right arm weakness remained.	First reported air embolism stroke case in Goldenhar syndrome surgery. Air embolism is rare but potentially life-threatening. Requires prompt recognition and multidisciplinary approach.
Pelo [67] 2009	To evaluate the outcome of segmental Le Fort I osteotomy with interpositional bone grafting for treating severe unilateral maxillary atrophy.	5 patients with severe unilateral maxillary atrophy. ICBG placed between maxillary fragments. Titanium fixation. Cancellous chips for sinus lift. On-lay grafts for transversal correction. Vestibuloplasty at 7 weeks. Second surgery at 4 months: removal of hardware and placement of 25 implants in 5 patients. Follow-up: minimum 1 year.	All cases successful. Correct intermaxillary relationship achieved. Inter-arch distance reduced. 25 implants placed, 1 lost after 1 year (96% success). All patients received partial-arch fixed prostheses.	Segmental Le Fort I osteotomy with bone grafting is safe and predictable for vertical and horizontal maxillary augmentation.
Posnick [68] 2015	This study evaluated safety and adequacy of re-harvesting anterior ICBG in young adults with repaired cleft undergoing Le Fort I advancement.	A retrospective review included patients under 26 who previously underwent ICBG and later required re-harvesting during Le Fort I surgery. Healing at donor and recipient sites was examined, and a questionnaire measured early and late discomfort.	Twenty-seven patients were included. All procedures yielded sufficient graft, and no complications occurred. Most patients reported easier recovery than after the first harvest, with no nerve injury or long-term functional issues.	Re-harvesting the anterior ICBG appears safe and effective for managing Le Fort I interpositional defects, providing adequate graft with minimal morbidity.
Bayat [69] 2011	To describe a comprehensive oral rehabilitation approach for hypohidrotic ectodermal dysplasia with severe oligodontia and maxillofacial hypoplasia using orthognathic surgery, bone grafting, and implants.	18 years old male with severe jaw atrophy, class III malocclusion, and single maxillary tooth treated in stages: (1) Le Fort I with bilateral sinus grafts ICBG/Bio-Oss 1:1, (2) iliac onlay grafts at 6 months, (3) placement of 14 implants (7 maxillary, 7 mandibular) at 30 N/cm torque.	Post-augmentation imaging confirmed a posterior maxillary bone height of roughly 22 mm. One mandibular implant failed during early healing but was subsequently replaced. At the 24-month review, all implants demonstrated clinical stability, preserved crestal bone levels, healthy peri-implant tissues, and stable occlusion. Maxillary repositioning remained unchanged, and no relapse was detectable.	The staged combination of orthognathic realignment, sinus lifting, and ridge augmentation can re-establish adequate bone volume for implant-supported rehabilitation in patients with ectodermal dysplasia. Over two years of follow-up, implant performance remained favorable, supporting their reliability as a long-term treatment option in individuals with severe congenital hypodontia.
Sabuncuoglu [70] 2010	To demonstrate ICBG combined with bimaxillary surgery for correcting severe paranasal hollowing in midface deficiency.	A 37-year-old woman with a skeletal Class III (ANB −12°, NV-A −4 mm, NV-Pog 17 mm) and deep bilateral paranasal depressions underwent presurgical orthodontics, Le Fort I advancement (6 mm), BSSO setback (7 mm), and ICBG (1 × 3 cm) fixed with 15 mm screws to augment paranasal regions. Pre/postop cephalometry and photos documented outcomes.	At 12-month follow-up, grafts showed complete integration with no complications. Skeletal correction: SNB 87° → 78°, SNA 75° → 80°, NV-Pog 17 mm → 0 mm (10 mm sagittal improvement). Occlusion and facial aesthetics significantly improved.	Combined orthognathic surgery with ICBG effectively corrects paranasal defects and midfacial contour, providing predictable structural support and aesthetic outcomes. Long-term monitoring recommended.
Piecuch [71] 1984	To assess outcomes and stability of maxillary augmentation via Le Fort I with interpositional ICBG for prosthetic rehabilitation.	3 patients with severe maxillary atrophy. Treatment: LeFort I osteotomy + cortico-cancellous ICBG. Follow-up with sequential lateral cephalometric radiographs up to 26 months.	Improved ridge height and dentures functional in all cases. Vertical relapse occurred mainly within the first 6 months. No secondary vestibuloplasty required.	Le Fort I osteotomy with interpositional grafts provides stable and predictable augmentation. Enables successful prosthetic rehabilitation in case of severe maxillary atrophy. Long-term success depends on coordinated surgeon-prosthodontist management.
Soehardi [72] 20152/23/2026 6:21:00 PM	The study aimed to examine long-term effects of a 2-stage reconstruction protocol using Le Fort I osteotomy with ICBG.	24 patients with severe maxillary atrophy (Cawood–Howell VI) treated with Le Fort I interpositional grafting, then implants at 3–6 months. Long-term follow-up evaluated bone levels, maxillary position, implant success, and patient satisfaction.	Healing was uneventful; 7 patients had minor bone defects at implant placement. 34/135 implants failed (screw-type superior); 2 patients lost all implants (multiple risk factors). Maxillary position stable with good functional outcomes reported.	The method provides stable reconstruction for advanced maxillary atrophy. Despite some implant failures, functional outcomes and patient satisfaction remain high. Newer implants designs may further improve long-term results.
Yerit [73] 2004	To evaluate long-term clinical and radiographic outcomes of horseshoe Le Fort I osteotomy with ICBG for severe maxillary atrophy reconstruction and implant rehabilitation.	36 patients with severe maxillary atrophy underwent HLFO with ICBG. Immediate group: 12 patients, 100 implants placed during surgery. Delayed group: 18/24 patients, 176 implants placed later. Follow-up assessed implant stability, bone height, peri-implant status, and functional/aesthetic outcomes.	2-year implant survival: 95.5%; 5-year: 89.3%. One-stage: 95.9%/86.9%; two-stage: 95.0%/91.3%. 27/276 implants failed (14 in 6 one-stage patients, 13 in 9 two-stage patients). Bone loss similar between groups. No significant survival difference (*p* = 0.57). All patients achieved improved aesthetics and function.	HLFO with ICBG proved effective for rebuilding the severely resorbed maxilla, offering stable implant survival and predictable functional and esthetic improvement. Both surgical approaches showed comparable long-term outcomes.
Wang [74] 2005	The study aimed to evaluate the effectiveness and stability of internal midface distraction in treating severe maxillary hypoplasia associated with cleft lip and/or palate.	10 patients with severe maxillary hypoplasia after cleft repair were treated using internal midface distractors. Various device types were applied. Six patients had an ICBG during Le Fort I osteotomy, and five with mandibular prognathism underwent bilateral sagittal split ramus osteotomy to improve facial balance and occlusion.	Maxillary advancement ranged from 5 to 15 mm, and the average SNA angle increased from 71.25° to 79.05°. New bone formed within the distraction gap, and follow-up confirmed stable maxillary position and occlusion without noticeable relapse.	Internal midface distraction provides effective maxillary advancement and stable long-term results in severe cleft-related maxillary hypoplasia.
Naros [75] 2019	To compare bone regeneration and stability after Le Fort I osteotomy using ICBG versus xenogenic bovine bone blocks for large defects.	25 patients underwent Le Fort I osteotomy and were randomized to receive either autogenous bone (*n* = 8) or bovine blocks in INTER (*n* = 12) or ONLAY (*n* = 5) positions. Histomorphometric biopsy analysis and serial cephalometric measurements were performed after healing.	Mineralized fraction: INTER 50.2 ± 13.2%, ONLAY 46.5 ± 12.3%, autogenous 57.1 ± 20.6%. New bone: 23.3 ± 14.1%, 14.9 ± 18.2%, 48.7 ± 27.8%, respectively. Maxillary advancement (SNA): INTER 3.6 ± 1.22°, ONLAY 4.7 ± 3.47°, BONE 2.4 ± 1.15°. Relapse rates: 20.5%, 30.3%, 33.0%.	Bovine bone blocks achieved mineralized fractions comparable to autogenous grafts. INTER positioning provided the lowest relapse rate. Xenogenic blocks represent a clinically effective alternative for large Le Fort I osteotomy gaps.
Varol [76] 2016	To assess implant survival and marginal bone loss after rehabilitation of severely atrophic maxillae (Cawood VI) using Le Fort I downgrafting with ICBG.	10 edentulous patients (mean age 50.4 ± 12.55 years) with Cawood VI atrophy underwent Le Fort I with ICBG (2009–2015). Mean advancement: 9 ± 1.4 mm; inferior repositioning: 8 ± 1.0 mm. 98 implants placed (80 maxillary, 18 mandibular) after 5.9 ± 0.73 months healing. Radiographic follow-up: 4 years.	Mean follow-up: 47.8 ± 3.4 months. Implant survival: 93.75% (9 failures). Marginal bone loss: 1.8 ± 1.0 mm at 1 year, 3.75 ± 0.85 mm at 4 years (*p* < 0.05). 1-year resorption significantly higher in 8-implant group vs. 6- and 10-implant groups (*p* = 0.045, *p* = 0.026).	Le Fort I downgrafting with ICBG achieves predictable long-term implant success in severe maxillary atrophy (Cawood VI), with high survival rates and acceptable bone loss over 4 years.
Isaksson [77] 1993	To assess early outcomes of simultaneous implant placement, bone grafting, and Le Fort I osteotomy in severe maxillary atrophy (Cawood VI).	12 patients (7 males, 5 females) with Cawood VI atrophy underwent Le Fort I with ICBG and immediate implant placement: 59 implants in grafted bone, 8 in native bone. Prosthetic loading at 9–12 months. Follow-up: 11–24 months.	Grafted bone: 14/59 implants failed (21% failure rate; mostly in 2 patients). Nongrafted bone: 8/8 implants succeeded. No implant loss post-loading. Hospital stay: 2–5 days. Minimal donor site morbidity; full ambulation within 1 month.	Immediate implant placement with Le Fort I and ICBG enables rehabilitation in severe maxillary atrophy, but requires high primary stability. Two-stage approach recommended when rigid fixation is unattainable to reduce early failure risk.
Nystrom [78] 1997	To evaluate outcomes of two-stage Le Fort I interpositional bone grafting for severe maxillary atrophy with delayed implant placement.	Ten edentulous patients (6 men, 4 women; mean age 50 years, range 38–58) with Cawood class VI maxillary atrophy underwent Le Fort I osteotomy with ICBG. Six months later, 60 titanium implants were placed. Follow-up ranged from 15 to 39 months after implant insertion.	Mean maxillary repositioning was 5 ± 3 mm horizontally and 5 ± 3 mm vertically. Relapse of 10–28% occurred in 6 patients during the first 6 months. Of 60 implants, 3 failed during initial healing (5% failure rate), with no further losses during a mean 27-month follow-up. All patients received fixed prostheses with full functional restoration.	Two-stage Le Fort I interpositional grafting reliably reconstructs severe maxillary atrophy with high implant survival and acceptable skeletal stability despite moderate early relapse.
Stork [79] 2013	To assess long-term skeletal stability and patient satisfaction after quadrangular Le Fort I osteotomy (QLF-I) with/without interpositional iliac grafts in cleft and non-cleft patients.	53 patients (mean age 18.6 ± 4.1 years; 60%F) from 212 treated (1984–2010) met criteria. 34 patients (64%) received ICBG, 19 did not. Serial cephalometry at T1, T2 (≤6 months), T3 (>12 months; mean 5.2 ± 5.2 y follow-up) assessed skeletal (A point, PNS) and dental (CI, 2 M) stability.	Mean advancement: 7.2 mm (A point), 7.7 mm (PNS). Horizontal relapse: 1.2 mm (A point), 0.9 mm (PNS); 86.5% had ≤3 mm relapse. Vertical augmentation: 3.1 mm with 1.4 mm relapse; impaction: 3.9 mm with 0.3 mm relapse. Patient satisfaction: 9.2/10; 95% reported improved occlusion.	Quadrangular Le Fort I osteotomy achieves high long-term skeletal stability in both planes with minimal relapse, even after large advancements. High patient satisfaction confirms this as a predictable technique for midfacial deficiency correction.
Nystrom [80] 2009	To evaluate long-term implant survival and marginal bone loss after Le Fort I interpositional grafting with delayed implants in severe maxillary atrophy, analyzing gender and smoking effects.	Twenty-six edentulous patients (13 men, 13 women; mean age 54.7 years, range 38–70) with Cawood class VI maxillary atrophy underwent two-stage Le Fort I osteotomy with ICBG. A total of 167 Brånemark implants were placed after 6 months of graft healing. Mean follow-up was 13 years (range 11–16 years).	Twenty-four of 167 implants failed (19 early, 5 late), yielding a cumulative survival rate of 85% at 10 years. Mean marginal bone loss reached 2.5 mm at 1 year, 2.9 mm at 2 years, and 3.1 mm at 10 years, with stabilization after 2 years. No significant differences in implant survival were found between smokers and non-smokers (86.1% vs. 84.6%) or between genders (*p* > 0.05).	Le Fort I interpositional grafting provides stable long-term implant survival and limited marginal bone loss in severe maxillary atrophy, with bone stabilization after 2 years, enabling predictable functional and aesthetic rehabilitation over > 10 years.
Van der Mark [81] 2011	To compare implant survival after severe maxillary atrophy reconstruction using Le Fort I interpositional grafting versus sinus floor elevation with onlay grafts.	27 patients (mean edentulism: 17.5 y, range 12–22) treated 2004–2007. Le Fort I group: 10 patients (5 M/5 F, age 53 ± 8 y) with ICBG. Sinus lift group: 17 patients (2 M/15 F, age 53 ± 7 y) with onlay grafts. 140 implants placed at 5–6 months post-grafting. Follow-up: 7–36 months.	Le Fort I group: 3/54 implants failed (6%). Onlay group: 2/86 failed (2%). Overall survival: 135/140 (96%); no significant difference (*p* = 0.3). Uneventful healing in all patients; no post-loading failures.	Both techniques achieve comparable high short-term implant survival in severe maxillary atrophy. Selection between Le Fort I and sinus lift with onlay grafts should prioritize interarch relationships and soft tissue needs over survival rates.
Kretschmer [94] 2010	To assess impact of additional procedures and ICBG on intraoperative blood loss during bimaxillary orthognathic surgery with multisegmental Le Fort I osteotomies.	225 patients (134 females, 91 males; mean age 26 y, range 16–54) underwent bimaxillary surgery with multisegmental Le Fort I (2006–2009). 49 received ICBG; 64 had additional osteotomies. Hemoglobin and PCV measured preoperatively and postoperatively (day 1).	Mean operative time: 258 ± 57 min. Postop hemoglobin decreased 25% (range 3–57%); PCV decreased 26% (−3% to 58%). Additional procedures caused significantly greater reductions (*p* < 0.001). 4 patients (2%) required transfusion, all from additional-procedure group.	Additional procedures with multisegmental Le Fort I significantly increase blood loss; operative time strongly correlates with hematological reduction. Despite this, transfusion rate remains low (2%), not justifying routine preoperative blood donation.
Marchetti [82] 2008	To compare long-term implant survival, success, and marginal bone loss in severely atrophic maxillae reconstructed with Le Fort I interpositional ICBG using machined versus TPS-surface implants.	12 edentulous patients (7 females, 5 males; age 55.1 ± 5.1 y, range 47–63) with Cawood VI atrophy underwent Le Fort I with ICBG. 104 implants placed at 5–6 months (53 machined, 51 TPS). Follow-up: 72–144 months (mean 102 ± 24.4 months).	11/104 implants failed; cumulative survival: 89.4% (machined 86.8%, TPS 92.2%; NS). Success rate: 67.3% overall (machined 66.0%, TPS 68.7%). Mean marginal bone loss: machined 2.91 ± 0.77 mm, TPS 2.72 ± 0.84 mm (NS).	Le Fort I interpositional grafting enables predictable long-term implant rehabilitation (6–12 years) in severe maxillary atrophy. Implant surface type (machined vs. TPS) does not significantly affect survival, success, or bone loss. Stable functional and aesthetic outcomes maintained despite progressive remodeling.
Sjostrom [83] 2006	To histomorphometrically compare implant integration in Le Fort I interpositional grafts versus onlay/inlay grafts, and assess timing of implant placement (simultaneous vs. delayed).	23 patients (14 females, 9 males) with severe maxillary atrophy: interpositional grafts (*n* = 8, age 54 y) or onlay/inlay grafts (*n* = 15, age 56 y). 68 titanium microimplants placed simultaneously or at 6 months, retrieved at 6–14 months. 3 control microimplants in nongrafted bone.	No significant differences between interpositional and onlay/inlay grafts in bone-implant contact, bone area in threads, or new bone (e.g., delayed bone-implant contact: 37.7 ± 20.5% vs. 25.2 ± 1.5%, *p* = 0.214). Delayed placement showed significantly higher bone-implant contact and new bone than simultaneous (*p* < 0.05). Nongrafted bone had significantly higher bone in threads (58.0 ± 13.7% vs. 25.7 ± 11.0%, *p* = 0.003) and new bone (75.0 ± 8.0% vs. 56.5 ± 10.9%, *p* = 0.009).	Implant integration is comparable between interpositional and onlay/inlay grafting techniques. Delayed implant placement after a 6-month healing period results in superior osseointegration compared with simultaneous placement. Timing of implantation plays a more critical role than the grafting technique itself.
Chiapasco [84] 2007	To evaluate the clinical outcome of osseointegrated implants placed in severely atrophied edentulous maxillae after Le Fort I osteotomy with interpositional ICBG	39 patients (18 males/21 females, age 32–76, mean 53.3 y) with severe maxillary atrophy (Class VI) from 3 centers (1995–2004). Le Fort I with ICBG and titanium fixation. Additional procedures: 2 BSSO setbacks, 5 buccal onlay grafts. 281 implants (4–10/patient) placed at 4–8 months. Prosthetic loading at 4–8 months: 19 fixed, 20 overdentures. Mean follow-up: 45.9 months (12–108 range)	38/39 reconstructions successful. 6 patients (42 implants) dropped out. 15 implants removed (5 pre-loading, 10 post-loading 1–3 years). 32 implants: excessive bone resorption but integrated. Survival rate: 94.5%. Success rate: 82.9%. Clinical parameters comparable to native bone. Patient satisfaction: 92.3%. Highest resorption within 3 years, then stabilized.	Le Fort I osteotomy with interpositional grafts and delayed implants is acceptable for severe maxillary atrophy. S
Posnick [85] 1994	Assessment of long-term skeletal stability and recurrence patterns in patients with unilateral cleft lip and palate who underwent Le Fort I surgery.	35 cleft lip/palate patients (mean age 18 y, range 14–25) underwent modified Le Fort I with ICBG and miniplate fixation (1987–1990). 24 maxillary-only, 11 bimaxillary procedures; 13 had concurrent pharyngoplasty. Intermaxillary fixation 6 weeks, splint 8 weeks. Pureed diet. Follow-up: 1.5–4.5 y (mean 2.5 y).	Mean horizontal advancement: 6.9 mm; maintained 5.3 mm at 1 y (relapse 1.6 mm). Vertical change: 2.1 mm initially, 1.7 mm at 1 y (relapse 0.4 mm). Positive overjet in all; positive overbite in 86% (30/35). No relapse difference between maxillary-only vs. bimaxillary (*p* = 0.05). Pharyngoplasty group: greater advancement (8.2 mm vs. 6.4 mm), similar relapse.	Miniplate fixation in Le Fort I for unilateral cleft patients provides acceptable stability with 6.9 mm horizontal advancement and 1.6 mm relapse. Pharyngoplasty does not significantly affect relapse despite larger initial advancement.
Eskenazi [86] 1992	To analyze Le Fort I maxillary advancement in cleft lip and palate patients comparing two fixation methods (wire vs. miniplate).	24 cleft patients (age 16–46, mean 27 years): 12 wire fixation, 12 miniplate fixation. Each group: 10 unilateral, 2 bilateral clefts. Modified high-level Le Fort I with autogenous grafts (iliac *n* = 14, mandibular *n* = 2, cranial *n* = 8). Onlay grafts for contour in selected cases. Wire group: intermaxillary fixation → elastics at 24 h → 3 weeks max. Miniplate group: rigid fixation, 6–8 wks wiring. Preop: 1–2 y orthodontics, alveolar grafting (*n* = 18).	Mean advancement: 7.8 mm horizontal (range 3 mm–2 cm), 2.3 mm vertical lengthening (range −5 mm to +1.5 cm). Wire group: 6.3 mm/0.8 mm initial → 4.2 mm/−0.5 mm at 1 y (33%/62% relapse). Plated group: 7.8 mm/4.3 mm → 7.5 mm/4.1 mm at 1 year (4%/5% relapse). Plated group significantly more stable; wire group showed significant dental relapse within 1 year.	Miniplate fixation superior to wire fixation for maxillary advancement stability in cleft patients (4–5% vs. 33–62% relapse). Relapse occurred mainly within first postoperative year (wire group). Pharyngeal flaps increase relapse tendency. Miniplate fixation with autogenous grafting provides sufficient stability without pterygomaxillary grafts.
Kahnberg [92] 1999	To evaluate combined treatment with Le Fort I osteotomy, interpositional bone grafts, and delayed implant placement.	25 patients (17 F, 8 M, mean age 56 y, range 38–77): development group (*n* = 5, 7 years follow-up), routine group (*n* = 20, up to 5 y). Inclusion: severe atrophy (Cawood–Howell IV–VI), 1–4 mm alveolar height, sagittal discrepancy. Stage 1: Le Fort I with ICBG (cortical/cancellous) in sinuses/nasal cavity, wire/miniplate fixation, 3–4 mo healing. Stage 2: plate removal, 181 Brånemark implants (6–8/patient) placed with guide splint, 6 mo healing. Temporary acrylic prosthesis 6 mo. 3 smokers quit 9 mo preoperatively.	Initial graft healing uncomplicated. 6 patients had sinus infections; 2 needed exploration for loose fragments. Iliac crest pain 1–2 weeks common. 30/181 implants lost: 14 at abutment connection/initial prosthetics, 16 between 1–4 y. Losses concentrated in 3 patients (15 implants); 14 had no losses. Final prostheses: 22 fixed, 2 overdentures, 1 prong denture.	Le Fort I with interpositional ICBG and delayed implant placement reliably reconstructs severe maxillary atrophy (Types IV–VI), correcting horizontal/vertical discrepancies. Two-stage protocol safe. Routine group: 85.6% 5-year implant survival. Recommend initial acrylic prosthesis to moderate forces during 1–2 y graft maturation. Smoking may negatively affect outcomes.
Farmand [87] 1986	To describe and evaluate horse-shoe sandwich osteotomy procedure for treating severe maxillary atrophy	15 patients (5 M, 10 F, mean age 52 y, range 36–65), 1990–1997. Indications: severe maxillary atrophy (13 with retromaxillism). Horse-shoe alveolar segment mobilized inferior/anteriorly via splint. Interpositional grafts: iliac crest (*n* = 13, L-shaped blocks) or ribs (*n* = 2). Osteosynthesis wires. Simultaneous vestibuloplasty when mucosa adequate. Temporary prosthesis day 7, definitive at 2 mo. Follow-up: 3–15 mo.	3 cases: maxilla advanced/lowered ~10 mm. 2 cases: inferior positioning only. Complications minimal: 3 superficial mucosal necrosis (scarring), 2 small oro-nasal fistulae (closed). No fractures of alveolar process or palatal roof-septum junction. Palatal height: increased in 13 patients, minimal in 2 (thick palatal mucosa).	Horse-shoe sandwich osteotomy avoids disadvantages of direct augmentation and standard Le Fort I. Single-session procedure increases vestibular and palatal height while improving mucosal contour. Little bone resorption observed (1–2 mm average). May be method of choice for extreme maxillary atrophy.
De Santis [93] 2012	Evaluation of the applicability of guided bone regeneration (GBR) using barrier membranes in Le Fort I osteotomy with interpositional bone grafts.	20 patients (5 M, 15 F, mean age 58.9 y, range 43–81) with severe atrophy (Cawood–Howell VI), unfavorable intermaxillary relations, ≥4 y follow-up. Exclusions: heavy smoking (>20 cigs/day), systemic disease, scarred tissues. Stage 1: Le Fort I with iliac cortico-cancellous blocks, cancellous/Bio-oss packing, Bio-Gide membrane coverage. Stage 2 (4 mo): 154 implants with surgical guide. Stage 3 (4 mo): healing abutments, temporary acrylic prosthesis 6 mo, then definitive fixed full-arch.	20/20 Le Fort I successful. 18 maxillary-only; 2 required BSSO; 6 had vestibular onlay. Mean advancement: 4.2 ± 0.5 cm (range 3.1–5 cm), stable at 4–6 y (4.1 ± 0.4 cm). Complications: iliac pain 1–2 wks common, 2 soft tissue dehiscences, no infections/sequestration. Implants: 152/154 osseointegrated; 2 failed integration. After loading: 2 bone loss (overload), 2 peri-implantitis (treated). Total failures: 6/154 in 5 patients. Success: 96.1%; cumulative 95.8%. Mean bone loss: 1.3 ± 0.4 mm (0.8–2.4 mm)	Le Fort I with interpositional grafts covered by barrier membranes predictably treats severe maxillary atrophy, compensating sagittal/vertical discrepancies with minimal resorption. GBR protocol achieves 95.8% implant success—comparable to native bone, superior to conventional Le Fort I—by preventing soft tissue interference during bone remodeling.
Sjostrom [88] 2005	To compare implants placed in grafted and normal non-grafted maxilla using resonance frequency analysis (RFA).	39 patients. Grafted group: 29 (21 females, 8 males, age 58 y, range 48–73) with severe atrophy (Cawood–Howell IV–VI). Stage 1:ICBG, 6 mo healing. Techniques: (1) onlay/nasal inlay (*n* = 24; 6 with sinus grafts, 18 posterior onlay); (2) Le Fort I interpositional (*n* = 5, reversed intermaxillary). Stage 2 (6 mo): 222 Brånemark implants (193 Standard, 29 Mark II; 10–18 mm × 3.75 mm, final drill 2.85 mm). Stage 3 (6–8 mo): abutment connection. Control: 10 non-grafted, 75 implants (final drill 3 mm). RFA/ISQ measured at: placement, abutment connection, 6 mo post-loading.	28/29 grafted patients completed (1 refused final). RFA on 212/222 implants. Failures: grafted 17/222 (8%; 13 early, 4 post-loading), non-grafted 1/75 (1%). Overall survival: 92%. RFA values (ISQ): Grafted: placement 61.5 ± 9.0, abutment 60.2 ± 6.9, loaded 62.5 ± 5.2 (*p* = 0.045 loaded vs. abutment). Non-grafted: placement 58.5 ± 4.7, abutment 60.9 ± 4.3, loaded 63.0 ± 5.6 (*p* = 0.022 loaded vs. placement). No significant difference between groups; both showed increasing stability over time.	Two-stage implants in grafted bone achieve stability comparable to non-grafted bone. RFA shows similar pattern in both groups with increasing stability over time. Six-month graft healing allows revascularization, enabling healing similar to native bone. All implants reach similar stability after 6 months loading, regardless of initial differences.
Sipos [89] 2025	To evaluate postoperative complications and reoperation rates after Le Fort I using demineralized bone matrix (DBM) versus autogenous bone grafts (ABG) in orofacial cleft and craniofacial malformation patients.	138 consecutive patients. DBM group (*n* = 103) received DBX, ABG group (*n* = 35) received ICBG. DBM: 53 females, 50 males, mean age 20.5 years. ABG: 19 females, 16 males, mean age 20.4 years. Diagnoses: Total cleft patients 113 (88 DBM, 25 ABG), craniofacial malformations/syndromes 25 (15 DBM, 10 ABG). Surgical procedures: Le Fort I osteotomy (84.8% cases), bimaxillary osteotomy (16.0% cases).	No statistical differences: age (*p* = 0.939), sex (*p* = 0.772), diagnosis (*p* = 0.063) between groups. DBM: 13.6% patients with complications (14/103), 17.5% overall complication rate. ABG: 20.0% patients with complications (7/35), 22.9% overall complication rate. Reoperation rate—Total: 6.5% (9/138). DBM: 6.8% (7/103). ABG: 5.7% (2/35)	No significant difference in complication or reoperation rates between DBM and ABG in maxillary osteotomies for cleft/craniofacial patients. DBM is a viable alternative offering: ready availability, no donor site morbidity, ~30 min shorter anesthesia, 30% lower cost, easier recovery.
Posnik [90] 1990	To investigate long-term skeletal stability after Le Fort I maxillary advancement in unilateral cleft lip and palate patients.	30 adults (mean age 18.0 y, range 13.4–23.3). All had Le Fort I; 15 bimaxillary (3 BSSO setback, 5 vertical genioplasty, 7 vertical genioplasty ± advancement). Grafts: iliac (*n* = 11) for residual clefts/interpositional/onlay; rib (*n* = 19, inframammary). Fixation: wire/intermaxillary 6–8 wks (*n* = 25), miniplates (*n* = 5).	Mean horizontal advancement: 1 wk 6.7 ± 2.6 mm (1.7–12.2 mm) → 6 wks 5.7 ± 2.5 mm → 1 y 4.9 ± 2.6 mm → 2 y 4.8 ± 2.6 mm (1.0–10.7 mm). Mean relapse at 2 y: 2.0 mm (0–3.7 mm). Wire group (*n* = 25): 1 wk 6.5 ± 2.7 mm → 1 y 4.6 ± 2.7 mm, relapse 1.9 mm. Miniplate (*n* = 5): 1 wk 8.0 ± 1.7 mm → 1 y 6.4 ± 2.2 mm, relapse 1.6 mm.	Le Fort I in unilateral cleft patients shows horizontal relapse primarily in first year, then stable. Mean 1.4 mm relapse at 2 years. Miniplate fixation achieved greater advancement but similar relapse. No significant differences (*p* > 0.05) between: maxillary-only vs. bimaxillary, iliac vs. rib grafts, segmentalized vs. non-segmentalized, with/without perioperative orthodontics.

ABG—autogenous bone graft, ANB—A point–Nasion–B point angle, BSSO—bilateral sagittal split osteotomy, CI—central incisor, DBM—demineralized bone matrix, DBX—demineralized bone matrix product, EBL—estimated blood loss, F—female, GBR—guided bone regeneration, HLFO—horseshoe Le Fort I osteotomy, ICBG—iliac crest bone graft, INTER—interpositional, ISQ—implant stability quotient, M—male, mo—month(s), NS—not significant, NV-A—Nasion vertical to point A, NV-Pog—Nasion vertical to pogonion, ONLAY—onlay, PCV—packed cell volume, PNS—posterior nasal spine, postop—postoperative, QLF-I—quadrangular Le Fort I osteotomy, RFA—resonance frequency analysis, SNA—sella–nasion–A point angle, SNB—sella–nasion–B point angle, T1/T2/T3—time points 1/2/3, TPS—titanium plasma-sprayed, wk—week, wks—weeks, y—year(s).

**Table 2:** Detailed characteristics of included studies.

Study	Study Type/Number of Patients	Maxillary Advancement (mm)	Type/Size of ICBG	Follow-Up Duration	Primary Outcomes	Complications
Pombo Castro [66], 2013	Case study, 1 patient	Data not included in the report	Autologous onlay and inlay ICBG (corticocancellous blocks for the Le Fort osteotomy and cancellous bone for sinus lift).	2 years	Balanced occlusion, Unilateral chewing (protecting the non-working side of TMJ) Satisfying Aesthetic result with lip support improvement, better self-esteem for the patient	Partial graft exposure with suppuration, which were removed under local anesthesia (patient smoked during the healing process)
Popat [91] 2024	Case presentation, 1 patient	Data not included in the report	anterior ICBG	Data not included for surgical outcomes, 3 months with the neurological assessment	Data not included for surgical outcomes	Data not included for surgical outcomes; air embolism treated conservatively
Pelo [67] 2009	Study of 5 patients with severe unilateral atrophy of the maxilla	Data not included in the report	cortical-cancellous bone graft from the anterior portion of the iliac crest and chips of cancellous bone (for sinus lift and onlay bone grafts)	Data not included in the study.	Achieved: improved maxillary contour with firm augmented ridges, corrected intermaxillary relationship and inter-arch distance, restored bone volume for implant placement (24/25 successful), adequate vertical augmentation and palatal-buccal dimension, corrected caudal fragment rotation via buccal onlay grafts.	Vestibuloplasty about 7 weeks after surgery
Posnick [68] 2015	Retrospective studies, 27 patients	7.4 mm horizontal, 2.4 mm vertical (at the incisors), 2.6 mm transverse (at the first molars)	Interpositional block graft, corticocancellous bone from anterior iliac crest	Min. 12 months after surgery	Graft rigidly fixed at osteotomy site	none
Bayat [69] 2011	Case report, 1 patient	Data not included in the report	Onlay graft from iliac corticocancellous bone, sinus augmentation from mixture of iliac bone and Bio-Oss (1:1)	24 months after delivery of final restoration	Appropriate healing with the stable bone height (22 mm) in the posterior maxillary area, one implant failure (out of 14), better intermaxillary relationship and facial profile, good functional and aesthetic results for the patient	Replacement of failed implant
Sabuncuoglu [70] 2010	Case report, 1 patient	6 mm	ICBG from anterior ilium (particulated spongious grafts, corticocancellous block bone grafts)	12 months	improved overall facial balance, successful incorporation of iliac block grafts, postoperative normalization of the cephalometric variables, improved speech and self-esteem for the patient	none
Piecuch [71] 1984	Case study 3 patients	Inferior repositioning (vertical): anterior 9–14 mm, posterior 3–4 mm; horizontal advancement not quantified.	Interpositional autogenous iliac cortico-cancellous grafts plus cancellous strips; graft size was not specified.	Follow-up reached up to 26 months (individual: 18, 24, 26 months).	New bone: not mentioned. Relapse: minor early vertical. Stability: stable augmentation, good implant survival. Outcome: improved denture function/aesthetics. Fixation: transosseous wire.	Specific complications were not reported. No reoperation was described; notably no secondary vestibuloplasty was needed before denture construction.
Soehardi [72] 20152/23/2026 6:21:00 PM	Retrospective study 24 patients	Maxillary advancement was evaluated cephalometrically using changes in the SNA angle; linear measurements in millimetres were not reported.	Interpositional corticocancellous ICBG were used; block size was approximately 5 × 6 cm, supplemented with particulate bone.	The follow-up ranged from 5 to 18 years.	New bone: not reported. Relapse: none clinically relevant. Stability: 74.8% implant survival; stable maxillary position long-term. Outcome: markedly improved patient satisfaction and function. Fixation: microplates/screws; sinus/nasal floors sealed with cortical plates.	Early sinus bone defects occurred in seven patients and required secondary grafting; no major long-term surgical complications were reported.
Yerit [73] 2004	Retrospective case series 36 patients	Maxillary advancement was not reported in millimeters; it was determined by cephalometric analysis and model surgery.	A corticocancellous ICBG was used as an interpositional (sandwich) graft; graft size was not specified.	The average follow-up was 94 months for group A and 45 months for group B, with an overall mean of 62 months. The follow-up range across both groups was approximately 0.3 to 126 months.	New bone: not reported. Relapse: assessed as vertical bone loss—mean 3.67 ± 2.77 mm (canine), 4.42 ± 2.72 mm (molar). Implant survival: 2 y: 95.9% (one-stage) vs. 95.0% (two-stage); 5 y: 86.9% vs. 91.3%. Outcome: all patients reported improved mastication and facial aesthetics. Fixation: micro- and miniplates.	Complications were mostly minor; secondary surgery was occasionally required for soft-tissue management. 27 of 276 implants failed overall. Only small corrective procedures were required, with no major secondary surgery.
Wang [74] 2005	Case series 10 patients	5–15 mm (mean 10.8 mm; ~9.6 mm left, 11.9 mm right), measured on pre- and post-distraction cephalograms	The size and exact type of the ICBG were not reported.	Mean follow-up 38.4 months (from 4 to 69 months).	New bone: qualitatively assessed (dense new bone), no percentage. Relapse: none obvious; ~8% based on SNA change. Stability: stable maxillary position/occlusion; no implant data. Outcome: improved occlusion and soft-tissue profile. Fixation: miniplates spanning cleft with graft.	Complications included TMJ pain caused by condylar displacement requiring immediate reoperation, local infection and pain, mucosal dehiscence leading to partial graft bone loss, and mechanical failure of the distractor requiring replacement.
Naros [75] 2019	Prospective clinical study with random allocation; 25 patients undergoing Le Fort I osteotomy: BONE (*n* = 8), INTER Bio-Oss^®^ block (*n* = 12), ONLAY Bio-Oss^®^ block (*n* = 5).	Mean SNA change (T1–T2): INTER 3.58° ± 1.22°, ONLAY 4.70° ± 3.47°, BONE 2.35° ± 1.15°.	ICBG placed into the osteotomy gap; graft size not numerically specified (block graft adapted intraoperatively to the defect). Comparator: bovine Bio-Oss^®^ block in INTER or ONLAY configuration.	Mean healing/observation period to hardware removal and biopsy: 11.6 ± 5.7 months (minimum ≥ 6 months). Radiological evaluation performed at T1, T2, and T3.	Histomorphometric and cephalometric outcomes: –Mineralized fraction: INTER 50.2% ± 13.2, ONLAY 46.5% ± 12.3, BONE 57.1% ± 20.6 –New bone formation: INTER 23.3% ± 14.1, ONLAY 14.9% ± 18.2, BONE 48.7% ± 27.8 –Postoperative stability (relapse based on SNA): INTER 20.5%, ONLAY 30.3%, BONE 33.0%	One inter-group patient developed late abscess and pseudarthrosis
Varol [76] 2016	Retrospective clinical study; 10 patients	Mean maxillary advancement 9 ± 1.4 mm; mean inferior repositioning 8 ± 1.0 mm	ICBG harvested from anterior ilium (*n* = 7) and posterior ilium (*n* = 3); some grafts sculpted into a horseshoe-shaped interpositional block. Numerical graft dimensions not reported.	Mean follow-up 47.8 ± 3.4 months; mean graft healing period before implant placement 5.9 ± 0.73 months.	–Implant survival rate 93.75%; failure rate 6.25% after 4 years (9 implant losses) –Peri-implant marginal bone resorption: 1 year: 1.8 ± 1.0 mm, 4 years: 3.75 ± 0.85 mm –98 implants placed total; 80 in maxillae, 18 in mandibles; fixed full-arch prosthetic rehabilitation achieved.	Complications: 2 palatal suture fractures during downfracture, 1 iliac seroma (conservative management). Implant failures: 5 maxillary (after healing cap), 4 mandibular (poor hygiene). No graft loss from infection/non-union.
Isaksson [77] 1993	Prospective clinical case series; 12 patients	Maxilla repositioned anteroinferiorly (~10 mm) to correct sagittal and vertical discrepancies; exact mean advancement not numerically reported.	ICBG placed to the nasal floor and maxillary sinuses; three graft blocks measuring approximately 2 × 0.8 × 0.8 cm each were used per patient.	11–24 months after surgery	–59 implants placed in grafted areas and 8 implants in nongrafted bone –14 implants (21%) failed due to lack of osseointegration (all within the first postoperative year); 10 failures occurred in 2 patients	2 complete implant failures per patient in two cases due to inability to achieve rigid fixation;
Nystrom [78] 1997	Prospective clinical case series; 10 patients	Mean maxillary repositioning: ~5 ± 3 mm anteriorly and ~5 ± 3 mm inferiorly. Relapse during 6-month healing observed in six patients, 10–28%	Autogenous corticocancellous anterior ICBG, harvested as a block approximately 4 × 2 × 1.5 cm	subsequent clinical follow-up 15–39 months	–60 implants placed (6 per patient) –3 implants failed to osseointegrate during healing and were removed at abutment surgery	Donor-site morbidity generally mild: postoperative pain, discomfort and gait disturbance mostly resolved within 3 weeks; at 2 months, pain in 2, discomfort in 1, gait disturbance in 3 patients.
Stork [79] 2013	Retrospective cohort: 121 questionnaire respondents, 53 met cephalometric criteria; 34/53 received interpositional iliac grafts.	Mean horizontal advancement (A-point): 7.2 mm; relapse 1.2 mm (50% ≤ 1 mm, 86.5% ≤ 3 mm; some 3–5 mm). Vertical augmentation: 3.1 mm; relapse 1.4 mm. Impaction relapse: 0.3 mm.	Interpositional corticocancellous ICBG, numerical graft dimensions not reported	T1 preoperative → T2 ≤ 6 months postoperative → T3 > 12 months postoperative (long-term).	Skeletal stability/relapse: Horizontal (A-point): mean 1.2 mm; 50% ≤ 1 mm, 86.5% ≤ 3 mm; some 3–5 mm (mainly cleft). Vertical: augmentation relapse 1.4 mm (A-point); impaction relapse 0.3–0.6 mm. Outcome: Mean satisfaction 9.2/10.	–Sensory disturbance in some patients -Iliac crest donor site morbidity limited: –77% had difficulty ambulating ≤ 2 weeks –75% donor site pain ≤ 4–6 weeks
Nystrom [80] 2009	Prospective follow-up study 26 patients	Not reported	Interpositional corticocancellous ICBG, numerical graft dimensions not reported.	Mean follow-up 13 years (range 11–16 years).	167 implants; 24 failures. Survival: 90.4% (1 y), 88.6% (2 y), 86.2% (5 y), 84.7% (10 y). Marginal bone loss: 2.5 mm (1 y), 2.9 mm (2 y, *p* = 0.01 vs. 1 y), 3.0 mm (5 y), 3.1 mm (10 y); stabilized after 2 y.	Supplementary implant placement performed in 14 of 24 failed implant cases
Van der Mark [81] 2011	retrospective comparative clinical study; 27 patients Le Fort I interpositional graft group: 10 patients	Mean forward repositioning of the maxilla: 5 mm Range: 2–8 mm (SD 1.9 mm)	Interpositional corticocancellous iliac blocks + inlay sinus cortical closure (posterior iliac crest). Particulate iliac bone mixed 4:1 with Bio-Oss^®^. Graft dimensions not reported.	Implants placed 5–6 months after grafting. Follow-up after implant insertion: 7–36 months.	Implant outcomes (Le Fort I group) Number of implants: 54 Failed implants: 3-failure 6% Implant losses occurred within first postoperative months; no further failures during follow-up.	(Le Fort I group) –One palatal fracture intraoperatively, but advancement completed successfully –Temporary sensory disturbance or donor site pain in a few cases, resolving ≤ 3 months
Kretschmer [94] 2010	Retrospective cohort: 225 consecutive patients with bimaxillary orthognathic surgery (multisegmental Le Fort I).	Not reported	ICBG. Additional procedures group (*n* = 93): iliac grafts *n* = 49, additional osteotomies *n* = 44, both *n* = 20. No additional procedures: *n* = 132. Numerical graft dimensions not reported.	Clinical follow-up period up to 17 months (range 7–36 months)	Not reported	4 transfusions required (2%)
Marchetti [82] 2008	Retrospective clinical study; 12 patients	4–8 mm anterior advancement 2–4 mm inferior repositioning	ICBG placed bilaterally in maxillary sinuses + premaxillary buccal onlay grafts for contour/stability. Exact dimensions not reported.	Follow-up after prosthetic loading: 6–12 years Mean follow-up: 102 ± 24.42 months Machined implant group: 104 ± 26.53 months TPS group: 100 ± 18.06 months	104 implants (53 machined, 51 TPS); 11 failures. Cumulative survival: 89.4% (6–12 y); machined 86.8%, TPS 92.2% (NS). Success: 67.3% overall (machined 66.0%, TPS 68.7%). MBR: machined 2.91 ± 0.77 mm (0.6–4.9 mm), TPS 2.72 ± 0.84 mm (0.7–5.3 mm); NS.	-Late infection in 2 patients (after 4–6 weeks) requiring removal of one miniplate each -Localized graft resorption at plate removal site but did not prevent implant placement
Sjostrom [83] 2006	Prospective histomorphometric clinical study; 23 patients Interpositional bone graft (IBG) after Le Fort I: *n* = 8 Onlay/inlay bone graft (OBG): *n* = 15	Not reported	ICBG: Interpositional corticocancellous grafts (Le Fort I group) Onlay + nasal floor inlay grafts, with additional sinus inlay grafts in 9 patients Numerical graft dimensions not reported.	Histomorphometry via scheduled titanium microimplant retrieval: simultaneous → 6 mo, simultaneous → 12–14 mo, delayed (after 6 mo graft healing) → 6–8 mo.	Bone-implant contact (BIC %) Interpositional vs. Onlay/Inlay: A (simultaneous, 6 mo): 14.6 ± 8.2 vs. 20.8 ± 17.6; B (simultaneous, 12–14 mo): 28.4 ± 16.7 vs. 23.2 ± 8.8; C (delayed, ≥6 mo): 37.7 ± 20.5 vs. 25.2 ± 1.5. No difference IBG vs. OBG; delayed higher than simultaneous (*p* < 0.05). Bone in threads (%): A: 22.1 ± 9.2 vs. 25.2 ± 17.9; B: 33.5 ± 16.7 vs. 24.0 ± 9.0; C: 41.8 ± 22.3 vs. 28.1 ± 17.6. NFB (%): A: 57.5 ± 7.1 vs. 63.2 ± 9.0; B: 67.3 ± 9.1 vs. 60.2 ± 8.6; C: 75.3 ± 12.3 vs. 66.3 ± 14.3. Nongrafted vs. grafted: Bone in threads 58.0 ± 13.7 vs. 25.7 ± 11.0 (*p* = 0.003); NFB 75.0 ± 8.0 vs. 56.5 ± 10.9 (*p* = 0.009).	–3 microimplants damaged during retrieval/processing –A few microimplants showed marginal bone resorption; majority showed none or minor
Chiapasco [84] 2007	Clinical follow-up study 39 patients (18 males, 21 females), aged: 32–76 years (mean: 53.3 years)	No data	Bicortical bone blocks: interpositional inlay shaped for anterior/lateral nasal floor and maxillary sinuses. Particulated bone filled remaining spaces. Buccal onlay grafts in 5 patients with extreme horizontal atrophy.	Mean follow-up: 45.9 months post-loading (range 12–108 mo; 1–9 y). Evaluated at: 1, 3, 6, 12 months post-loading, then annually.	New bone: 38/39 successful reconstruction (97.4%); 1 partial failure (graft exposure/infection), 2 buccal onlay resorptions. 281 implants (4–10/patient): 94.5% survival, 82.9% success; 15 removed (5 pre-loading, 10 post-loading). Patient satisfaction: 92.3%.	Reoperation: 25.6% (10/39). Graft-related: 1 resorption, 1 infection, 1 implant dehiscence. Implant-related: 3 patients/4 implants replaced; 7 patients/11 implants failed without replacement (prosthesis adapted).
Posnick [85] 1994	Prospective study 35 patients (mean age 18 years, range 14–25 years)	Horizontal: mean 6.9 mm initially achieved, 5.3 mm 1 year after, mean relapse: 1,6 mm horizontally Vertical: mean change 2,1 mm initially, 1.7 mm 1 year after, mean vertical relapse 0.4 mm	Corticocancellous grafts	Radiographic: preop, immediate postop (3–7 d), 6–8 wks, 1 y. Clinical follow-up: 1.5–4.5 y (mean 2.5 y), range up to 5 y (mean 1.5 y). Exclusions: 10 patients (7 incomplete records, 3 < 3 mm advancement).	Relapse: horizontal mean 1.6 mm, <1 mm in 11/35 patients (31%), vertical mean 0,4 mm stability: 100% positive overjet, 86% positive overbite (30/35)	Need for reoperation not reported
Eskenazi [86] 1992	Comparative study 24 patients (14–46 years, mean age 27)	Horizontal: Wire: 6.3 mm → 4.2 mm at 1 y, relapse 2.1 mm (33%). Miniplate: 7.8 mm → 7.5 mm at 1 y, relapse 0.3 mm (4%). Vertical: Wire: 0.8 mm → −0.5 mm at 1 y, relapse 1.3 mm (162%). Miniplate: 4.3 mm → 4.1 mm at 1 y, relapse 0.2 mm (5%).	Graft: cranial bone: 8 patients, iliac bone: 14 patients, mandibular bone: 2 patients (from chin)	Minimum: 1 y cephalometric follow-up. Long-term (>2 y): 20 patients. Radiographic intervals: preop, immediate postop, 6 mo, 1 y.	Relapse—miniplate group more stable in both dimensions Class I occlusion 1 year after in all patients	-1 patient: Required subsequent procedure to correct velopharyngeal insufficiency after maxillary advancement -4 patients: Developed incisor angulation requiring compensation (wire group) -3 patients: Transverse collapse requiring management (wire group)
Kahnberg [92] 1999	Prospective study 25 patients (17 females, 8 males; aged 38–77 years, mean 56 years)	Maximum forward repositioning: 10 mm Vertical correction: Achieved by rotating maxilla inferiorly	Graft: -cortical bone -cancellous bone -interpositional grafts	Follow-up: 6 patients 7 y, 6 patients 4 y, 4 patients 3 y, 4 patients 2 y, 3 patients 1 y, 2 patients < 1 y. Total 20 patients followed > 2 y.	Development: 60.0% (5 y). Routine group: 85.6% (5 y, life table). Total: 30/181 losses (16.6%): 14 at abutment/initial prosthetics, 16 between 1–4 y. Concentrated: 15/30 in 3 patients. 14/25 patients: no failures.	Sinus Infections -6 patients: sinus infections developed -2 patients (8%): Exploration and removal of loose bone fragments 1 patient: prong denture after losing 5/6 implants (heavy bruxer)
Farmand [87] 1986	Case series 15 patients (5 males, 10 females, 36–65 years, mean 52 years)	Advancement and lowering: 10 mm (13 patients) Vertical only: 2 patients (no advancement) Intraoperative vertical increase: 10 mm bony height	Graft: -ICBG (13), L-shaped cortical blocks -ribs (2), whole rib -interpositional (between mobilized alveolar process and midface)	Range: 3–15 months postoperatively	-100% improved facial appearance -vestibular height: 11/15 very good, 3/15 good and 1/15 moderate result -palatal height 13/15 achieved -bone resorption: 0–3 mm (average 1–2 mm) at first year range	-3 patients superficial mucosal necrosis (secondary suturing) -2 patients with small fistula (easily closed) -4 patients secondary vestibuloplasty
De Santis [93] 2012	Prospective study 20 patients (5 males, 15 females, 43–81 years, mean 58.9 years)	Mean advancement: 42 mm (4.2 cm) Range: 31–50 mm (3.1–5 cm) At 4–6 year follow-up: 41 mm (4.1 cm) maintained	Anterior iliac crest for all patients and posterior if larger volume needed Graft: -corticocancellous blocks: -cancellous fragments: Packed around blocks	Patient: mean 51.5 ± 9.3 mo (35–65 mo); ≥4 y: 100% (20/20), ≥5 y: 80% (16/20), ≥6 y: 30% (6/20). Implant: mean 66.4 ± 18.4 mo (48–74 mo); ≥4 y: 100% (152), ≥5 y: 81.6% (124), >6 y: 31.6% (48). No dropouts; all completed.	Le Fort I with 100% success, mean advancement maintained: 4.2 cm → 4.1 cm at 4–6 years. Bone resorption: mean peri-implant bone loss 1.3 ± 0.4 mm during loading (range 0.8–2.4 mm), implant success 96.1%	-1 patient peri-implantitis(reoperated) -2 patients overloading (adjustments) -2 patients with 2 implants failed integration (removed without replacement)
Sjostrom [88] 2005	Prospective study 29 patients (21 females, 8 males, 48–73 years, mean 58 years)	No measurements provided	-24 patients L-shaped onlay grafts -5 patients interpositional grafts	Implant healing: 6 mo (*n* = 10, standard); 8 mo (*n* = 19, prolonged due to low stability/previous failures/bruxism). Follow-up: minimum 1 y post-loading. RFA: 3 timepoints—placement, abutment connection (6–8 mo), 6 mo post-loading.	Grafted bone: 17/222 failed (8%); 92% survival (13 early, 4 late). Non-grafted: 1/75 failed (1%); 99% survival. Primary stability: Mobile implants (20/222, 9%): 52.8 ± 13.1 ISQ (*p* = 0.020); stable: 62.3 ± 10.4 ISQ. 7/20 mobile failed (35%). Failed vs. successful: 54.6 ± 12.0 vs. 62.0 ± 10.8 ISQ at placement (*p* = 0.072). Grafting: Interpositional (*n* = 5): 65.7→61.4→61.4 ISQ; Onlay (*n* = 24): 60.6→59.9→62.7 ISQ.	-1 patient graft resorption (reoperation) -implant failures: early—13 implants (4 replaced), late—4 implants lost after loading
Sipos [89] 2025	Retrospective study 138 patients (DBM 103 patients, ABG 35 patients, 72 females, 66 males)	No measurements provided	DBM group: demineralized bone matrix ABG: iliac crest (cortico-cancellous)	Time period: 2014–2022 (8 years)	Complication Rates -Overall: 18.8% -DBM group: 13.6% of patients (17.5% overall complication rate) -ABG group: 20.0% of patients (22.9% overall complication rate)	Reoperation Rates -overall: 9/138 (6.5%) -DBM group: 7/103 (6.8%) -ABG group: 2/35 (5.7%)
Posnik [90] 1990	Restrospective study 30 patients with unilateral cleft lip and palate	Horizontal: mean 4.8 mm at 2 years (range 1–10.7 mm), direct wire fixation: 6,5 mm (1 week), 4.6 mm (1 year); miniplate fixation: 8.0 mm (1 week), 6.4 mm (1 year) Vertical: mean downward: 2.6 mm immediately post-op; mean 1.2 mm at 1 year	ABG: all 30 patients. Anterior iliac crest: 11 patients, Rib graft: 19 patients.	Minimum 2 years postoperatively follow-up. Serial cephalometric radiographs at: preoperative, immediate post-op, 6–8 weeks, 1 year, and 2 years	Graft stability: no correlation between advancement and relapse, maxillae stable after first year	Not mentioned of complications or reoperations.

ABG—autogenous bone graft, BIC—bone-to-implant contact, Bio-Oss®—deproteinized bovine bone mineral, cm—centimeter(s), d—day(s), DBM—demineralized bone matrix, IBG—interpositional bone graft, ICBG—iliac crest bone graft, INTER—interpositional, ISQ—implant stability quotient, min.—minimum, mm—millimeter(s), mo—month(s), NFB—newly formed bone, NS—not significant, n—number of patients/specimens, OBG—onlay/inlay bone graft, ONLAY—onlay, postop—postoperative, preop—preoperative, RFA—resonance frequency analysis, SD—standard deviation, SNA—sella–nasion–A point angle, TMJ—temporomandibular joint, T1/T2/T3—time points 1/2/3, TPS—titanium plasma-sprayed, wk—week, wks—weeks, y—year(s).

**Table 3:** Quality Assessment.

Authors	1. Is the Sampling Strategy Relevant to Address the Research Question?	2. Is the Sample Representative of the Target Population?	3. Are the Measurements Appropriate?	4. Is the Risk of Nonresponse Bias Low?	5. Is the Statistical Analysis Appropriate to Answer the Research Question?
Pombo Castro [66], 2013	No	No	Yes	Yes	No
Popat [91] 2024	No	No	Yes	Yes	No
Pelo [67] 2009	No	No	Yes	Yes	No
Posnick [68] 2015	Yes	Yes	Yes	No	No
Bayat [69] 2011	No	No	Yes	Yes	No
Sabuncuoglu [70] 2010	No	No	Yes	Yes	No
Piecuch [71] 1984	No	No	Yes	Yes	No
Soehardi [72] 20152/23/2026 6:21:00 PM	Yes	Yes	Yes	Yes	Yes
Yerit [73] 2004	Yes	Yes	Yes	Yes	Yes
Wang [74] 2005	Yes	No	Yes	Yes	Yes
Naros [75] 2019	Yes	No	Yes	Yes	No
Varol [76] 2016	Yes	No	Yes	Yes	Yes
Isaksson [77] 1993	Yes	Yes	Yes	No	Yes
Nystrom [78] 1997	Yes	Yes	Yes	Yes	Yes
Stork [79] 2013	Yes	Yes	Yes	Yes	Yes
Nystrom [80] 2009	Yes	Yes	Yes	Yes	Yes
Van der Mark [81] 2011	Yes	Yes	Yes	Yes	Yes
Kretschmer [94] 2010	Yes	Yes	Yes	Yes	Yes
Marchetti [82] 2008	Yes	Yes	Yes	Yes	Yes
Sjostrom [83] 2006	Yes	Yes	Yes	Yes	Yes
Chiapasco [84] 2007	Yes	Yes	Yes	Yes	Yes
Posnick [85] 1994	Yes	Yes	Yes	No	Yes
Eskenazi [86] 1992	Yes	Yes	Yes	No	Yes
Kahnberg [92] 1999	Yes	Yes	Yes	Yes	Yes
Farmand [87] 1986	Yes	Yes	Yes	Yes	Yes
De Santis [93] 2012	Yes	Yes	Yes	Yes	Yes
Sjostrom [88] 2005	Yes	Yes	Yes	Yes	Yes
Sipos [89] 2025	Yes	Yes	Yes	Yes	Yes
Posnik [90] 1990	Yes	Yes	Yes	No	Yes

## Data Availability

Data supporting the findings of this study are available within the article.

## Supplementary Materials

The following supporting information can be downloaded at: https://www.mdpi.com/article/10.3390/jcm15072586/s1, PRISMA 2020 Checklist [55].
